# Rho family GTPase 1 (RND1), a novel regulator of p53, enhances ferroptosis in glioblastoma

**DOI:** 10.1186/s13578-022-00791-w

**Published:** 2022-05-03

**Authors:** Qian Sun, Yang Xu, Fan’en Yuan, Yangzhi Qi, Yixuan Wang, Qianxue Chen, Baohui Liu

**Affiliations:** 1 Department of Neurosurgery, Renmin Hospital of Wuhan University, Wuhan University, 238 Jiefang Street, Wuhan, Hubei 430060 People’s Republic of China; 2 Central Laboratory, Renmin Hospital of Wuhan University, Wuhan University, Wuhan, Hubei 430060 People’s Republic of China

**Keywords:** RND1, p53, Ferroptosis, GBM

## Abstract

**Background:**

Ferroptosis is an iron dependent cell death closely associated with p53 signaling pathway and is aberrantly regulated in glioblastoma (GBM), yet the underlying mechanism needs more exploration. Identifying new factors which regulate p53 and ferroptosis in GBM is essential for treatment.

**Methods:**

Glioma cell growth was evaluated by cell viability assays and colony formation assays. Lipid reactive oxygen species (ROS) assays, lipid peroxidation assays, glutathione assays, and transmission electron microscopy were used to assess the degree of cellular lipid peroxidation of GBM. The mechanisms of RND1 in regulation of p53 signaling were analyzed by RT-PCR, western blot, immunostaining, co-immunoprecipitation, ubiquitination assays and luciferase reporter assays. The GBM‐xenografted animal model was constructed and the tumor was captured by an In Vivo Imaging System (IVIS).

**Results:**

From the The Cancer Genome Atlas (TCGA) database, we summarized that Rho family GTPase 1 (RND1) expression was downregulated in GBM and predicted a better prognosis of patients with GBM. We observed that RND1 influenced the glioma cell growth in a ferroptosis-dependent manner when GBM cell lines U87 and A172 were treated with Ferrostatin-1 or Erastin. Mechanistically, we found that RND1 interacted with p53 and led to the de-ubiquitination of p53 protein. Furthermore, the overexpression of RND1 promoted the activity of p53-SLC7A11 signaling pathway, therefore inducing the lipid peroxidation and ferroptosis of GBM.

**Conclusions:**

We found that RND1, a novel controller of p53 protein and a positive regulator of p53 signaling pathway, enhanced the ferroptosis in GBM. This study may shed light on the understanding of ferroptosis in GBM cells and provide new therapeutic ideas for GBM.

**Supplementary Information:**

The online version contains supplementary material available at 10.1186/s13578-022-00791-w.

## Introduction

Glioblastoma (GBM) is a common intrinsic brain tumor and accounts for a high proportion of gliomas and has the highest mortality rate of all central nervous system tumors [1]. Despite surgical resection followed by radiotherapy and temozolomide (TMZ) therapy, the median survival is only raised to 14.6 months [2], indicating the urgent need for novel therapeutic strategies. Recent evidence suggests that ferroptosis plays a crucial role in various tumors and is aberrantly regulated in glioblastoma [3] and the ferroptosis-related gene signature predicts GBM prognosis [4]. The study of the regulatory mechanism and effective targets for ferroptosis may lead to new therapeutic strategies for GBM.

As a transcription factor, p53 mediates ferroptosis activity in cells during tumour suppression [5]. Solute carrier family 7 member 11 (SLC7A11), the core component of system x_c_^−^ which mediates the uptake of extracellular cystine in exchange for intracellular glutamate is transcriptionally downregulated by p53 [6]. Through modulation of SLC7A11, p53 leads to reactive oxygen species (ROS)–induced stress and sensitizes cells to ferroptosis. The ubiquitin–proteasome is known to play a significant role in the regulation of the p53 protein. The ubiquitin ligase represented by Mdm2 is the primary negative regulator of p53, which promotes p53 ubiquitination and translates into the degradation of p53 protein [7].

In contrast, the stabilization of p53 protein is adjusted by the deubiquitinating enzymes, which protect p53 from ubiquitination-mediated proteasomal degradation [8]. For instance, ubiquitin specific peptidase 7 (USP7), one of the deubiquitinases, was reported to stabilize p53 and stimulate its activity, thus leading to glioblastoma apoptosis [9]. Overall, the p53 protein is regulated in diverse ways, and p53 plays an important role in ferroptosis, but the studies related to GBM are insufficient.

Rho family GTPase 1 (RND1), an atypical member of the Rho GTPase family, is predominantly in an active GTP-bound conformation that lacks GTP hydrolysis activity [10]. The essential function of RND family, including RND1, is to regulate the morphology and cytoskeleton [11], while RND1 also participates in angiogenesis, nerve connections and embryonic development [12]. The current research indicated that RND1 displays the characteristics of a tumor suppressor gene. Okada et al. discovered that RND1 suppresses mammary tumorigenesis through the epithelial-to-mesenchymal transition (EMT) by restraining Ras-MAPK pathway [13]. Subsequently, RND1 was reported to inhibit EMT and serve as a positive prognostic factor in hepatocellular carcinoma [14, 15]. The role of RND1 in GBM was unknown until a study in 2018 demonstrated that glioma stem-like cells in the periventricular zone possess higher invasive capacities regulated by RND1 [16]. As a result, the function of RND1 in GBM remains unclear, and its regulatory mechanism requires elucidation.

This study aimed to clarify the signaling pathway through which RND1 mediates ferroptosis in GBM. The RND1 expression level was significantly downregulated in human GBM samples, suggesting a favorable prognosis of RND1 in GBM patients. Furthermore, we demonstrated that RND1 promoted GBM cell ferroptosis in vivo and in vitro*.* In addition, RND1 regulated the p53-SLC7A11 pathway by directly binding to the p53 protein and affecting p53 degradation. Our study identified a novel function of RND1 in GBM and provided mechanistic insights into regulating the p53-SLC7A11 pathway in ferroptosis.

## Materials and methods

### Human samples

Human glioma and normal brain samples were collected in the Department of Neurosurgery, Renmin Hospital of Wuhan University. Tumor tissues were obtained in the patients with diagnosed glioma which was later confirmed independently by three neuropathologists of Department of Pathology at Renmin Hospital of Wuhan University. Normal brain samples were collected during craniotomies of the patients with intracranial hypertension. The procurement and use of tissue in this study were approved by the Institutional Ethics Committee of the Faculty of Medicine at Renmin Hospital of Wuhan University (approval number: WDRY2015-K016) and performed with patient-informed consent. The clinical information of human samples was shown in Additional file 8: Table S2 and Additional file 9: Table S3.

### Reagents and antibodies

The following antibodies were used: anti-GAPDH (5174, Cell Signaling Technology), anti-SLC7A11 (26864-1-AP, Proteintech), anti-p53 (10442-1-AP, Proteintech), anti-GPX4 (A11243, ABclonal Technology), anti-ACSL4 (A6826, ABclonal Technology), anti-FTL (ab69090, Abcam), anti-NRF2 (M200-3, Medical Biological Laboratories), anti-ATF3 (P18847, Cusabio), anti-ATF4 (P18848, Cusabio), anti-HA (M180, Medical Biological Laboratories), anti-DDDDK (M185, Medical Biological Laboratories), anti-ub (10201-2-AP, Proteintech), anti-RND2 (GXT56070, GeneTex), anti-RND3 (GTX81316, GeneTex) and anti-Mouse IgG (A7028, Beyotime Biotechnology). Several anti-RND1 antibodies in the market has been tried which showed the poor sensitivity, and they were prone to non-specific reactions with other RND members, such as RND2 and RND3. We selected the latter part of the RND1 sequence (CSLSKRLLHLPSRSE) to predict the antigen peptide and synthesized an anti-RND1 antibody by Genscript (Nanjing, China). The specificity of RND1 antibody was verified (Additional file 1: Fig. S1B). DMSO was purchased from Servicebio (WD2650, Servicebio). The apoptosis inhibitor Z-VAD-FMK (S7023), the ferroptosis inhibitor Ferrostatin-1 (S7243), the necrosis inhibitor Necrosulfonamide (S8251) and the ferroptosis inducer Erastin (S7242) were purchased from Selleck (USA).

### Cell culture

Human GBM cell lines (U87, A172, U118 and U251) and human renal epithelial cell line (293T) were purchased from Shanghai Institute of Cell Biology, Chinese Academy of Sciences (Shanghai, China). Authentication of GBM cell lines was conducted via short tandem repeat profiling in Procell Life Science & Technology Co., Ltd. All cell lines were cultured in high-glucose DMEM (Genom, Hangzhou, China) containing 10% fetal bovine serum (Gibco, Thermo Fisher Scientific, USA), penicillin (100 U/mL) and streptomycin (100 µg/mL) at 37 °C in a humidified atmosphere containing 5% CO_2_.

### Generation of stable cell line and intracranial xenograft model

To establish the stable cell line, plasmids with lentiviral vector, pLVX-CMV-Luciferase-RND1 and pLVX-CMV-Luciferase were transfected with pMD2.G and psPAX2 into 293T cells to produce lentiviruses. U87 cells were infected with the lentiviruses and selected by puromycin at a dilution of 5 mg/mL.

Four-week-old male Balb/c nude mice (n = 30) were purchased from Vital River Laboratory Animal Technology (Beijing, China) and raised in specified pathogen-free (SPF) condition. 5% chloral hydrate was injected intraperitoneally for anesthesia. Intracranial injection of U87 cells into the lateral ventricle was described previously [17]. 30 nude mice were divided into the experimental group (U87-Luciferase-RND1, n = 14), the control group (U87-Luciferase, n = 14) and the sham operation group (PBS, n = 2). Tumor-bearing mice were captured by IVIS (In Vivo Imaging System) at the 21st day after tumor injection and sacrificed at their terminal point which was defined as severe neurological symptoms. Animal experiment of this research was approved by the institutional animal care and use committee of Renmin Hospital of Wuhan University.

### Bioinformatics

The expression profiles of RND1 in different normal tissues were downloaded from the HPA database, and the data of RND1 in different human tumors was searched from The Cancer Genome Atlas (TCGA) database. We used the Gliovis database to clarify the RND1 expression in different grades and subtypes of glioma (Table 1).

**Table 1:** Kaplan–Meier analysis of clinical GBM patients

Clinicopathologic variables	Number	Average survival	*P* value
All patients	45	16.005 ± 2.360	
Gender			
Male	24	13.786 ± 2.595	0.518
Female	21	18.087 ± 3.903	
Age at diagnosis			
< 55	24	19.163 ± 4.097	0.501
≥ 55	21	13.148 ± 1.726	
KPS			
< 70	11	8.273 ± 2.149	0.018*
≥ 70	34	16.005 ± 2.360	
Headache			
Yes	21	12.551 ± 1.532	0.775
No	24	17.104 ± 3.520	
Intracranial infection			
Yes	3	6 ± 4.041	0.05
No	42	16.752 ± 2.489	
Multiple lobe lesions			
Yes	11	15.697 ± 3.715	0.727
No	34	15.913 ± 2.815	
RND1 expression			
High	22	20.801 ± 3.912	0.007*
Low	23	10.947 ± 1.828	

### Western blot analysis

Cell samples were lysed in RIPA buffer (Beyotime, Shanghai, China) supplemented with a protease inhibitor Cocktail (Roche, Indianapolis, IN) and the protein concentration was measured by a BCA Kit (Beyotime, shanghai, China). Then, protein samples (20 μg/lane) were separated on SDS–polyacrylamide gels and transferred onto 0.45-mm PVDF membranes (Millipore, Germeny). After immunoblotted with primary antibodies at 4 °C overnight, the membranes were incubated with HRP-conjugated secondary antibody and captured using a ChemiDoc Imaging System (Bio-Rad, USA). Densitometry analysis was normalized to GAPDH.

### Co-immunoprecipitation (Co-IP) and ubiquitination assays

For the verification of protein interaction, 293T cells were co-transfected with Flag-RND1 and HA-p53 (Miaoling Biology) plasmids for 48 h. Cell samples were collected and lysed in IP buffer (50 mM Tris–Cl at pH 8.0, 0.5 mM EDTA, 150 mM NaCl, 1% NP-40, 50 mM NaF, 2 mM Na3VO4, 4 mM Na pyrophosphate) supplemented with 1 × Cocktail (Roche, Indianapolis, IN). The lysates were incubated with 3 μg of primary antibodies (anti-Flag-tag, anti-HA-tag and IgG) and Protein G Agarose (sc-2003, Santa Cruz Biotechnology, USA) overnight at 4 °C. The precipitates were collected by the centrifugation at a speed of 2500 rpm and washed 5 times with IP buffer. Bound proteins were eluted using 1.5 × loading buffer (Beyotime) and separated on SDS–polyacrylamide gels, followed by western blot analysis.

For the analysis of ubiquitination, 293T cells were co-transfected with the HA-p53 and His-ub-WT (Miaoling Biology), His-ub-K48R (Miaoling Biology) or His-ub-K63R (Miaoling Biology) plasmid. The lysates were precipitated by anti-HA and repeated the procedures as IP mentioned above. Bound proteins were immunoblotted by an anti-ub antibody.

### Immunohistochemical staining (IHC)

Brain tissues from human or mice were embedded with paraffin and sectioned. The sections were heated, deparaffinized, rehydrated and repaired with 10 mM sodium citrate (pH 6.0). We removed endogenous peroxidase using 3% H_2_O_2_ and blocked the sections with 1% bovine serum albumin (Amresco, USA). The slides were incubated with primary antibody at 4 °C overnight. The tissues were dyed by HRP-labelled secondary antibodies (Servicebio, China) and 3, 3′-diaminobenzidine (DAB) (Servicebio). The nuclei were stained by haematoxylin. The images were captured by an Olympus BX51 microscope (Olympus, Tokyo, Japan).

### Tissue microarray analysis

In the microarray analysis, we assessed the protein level of RND1, p53 and SLC7A11 in 53 GBM tissues according to the IHC procedure. The distribution and intensity of protein staining was first analyzed and described as negative, weak, moderate and strong. Tissues with negative and weak staining were classified in low expression group, while tissues with moderate and strong staining were classified in high expression group. Chi-square test was used to explore the correlation between RND1 and p53/SLC7A11.

### RNA isolation and RT-PCR

Total RNA of GBM cells were extracted using TRIzol regent (Invitrogen). RNA concentration was detected by a Nanodrop (Thermo Fisher Scientific, USA). We used PrimeScript RT reagent kit (Takara, Japan) for preparing cDNA and SYBR Green II Mixture (TaKaRa) for real-time PCR according to the manufacturer’s protocol. The comparative Ct method (ΔΔCt) was used to evaluate mRNA expression and GAPDH was used for normalization. Specific primer pairs were provided in Additional file 7: Table S1.

### DNA and shRNA construction of RND1

pCDNA3.1-Flag-RND1 and pLVX-CMV-RND1-P2A-Fluc-Puro plasmids were constructed by Miaoling Biology and the sequence of RND1 was provided in Additional file 7: Table S1. Specific shRNA targeting of human RND1 (shRND1) and negative control shRNA (shNC) were purchased from Miaoling Biology. The sequences of shRNA were provided in Additional file 7: Table S1. Transfections were helped with Lipofectamine 3000 (L3000015, Thermo Fisher Scientific).

### Dual-luciferase reporter assays

MG15-luciferase (mutated p53 binding sites) and PG13-luciferase (wild-type p53 binding sites) plasmids were constructed by Miaoling Biology. The pGMLR-TK luciferase reporter plasmid was purchased from Yeasen Technology (Shanghai, China) and used for renilla luciferase reporter as a control. In the reporter assay, cells were co-transfected with the wild-type or mutated p53 luciferase reporter plasmids accompanied by the renilla luciferase reporter plasmid. 48 h after the transfection, cells were collected and dealt with a Dual Luciferase Reporter Gene Assay Kit (Yeasen Technology). The luminescence from firefly and renilla luciferase was detected by a Multimode Plate Reader (PerkinElmer, Germany).

### Cell viability assays

Cell viability was measured by Cell Counting Kit-8 (CCK-8) (Dojindo Molecular Technologies, USA). Cells were seeded into 96-well plates (5000 cells/well) and detected at specified time points (0, 24, 48 and 72 h). After CCK8 regents (10 μl/well) were added and incubated at 37 °C for 1 h, the absorbance value was detected at 450 nm using a Multimode Plate Reader (PerkinElmer, Germany).

### Colony formation assays

For the colony-formation assay, 500 cells per well were seeded into six-well plates and cultured for three weeks. The colonies were fixed with 4% paraformaldehyde and stained by crystal viole. The representative colonies were captured and quantified.

### Lipid ROS assays

We analysed the lipid ROS of cells using flow cytometry. Cells were treated in six-well plates and harvested by trypsinization. The samples were incubated in PBS containing 2 μM C11-BODIPY (581/591) (#D3861, Invitrogen) for 30 min at 37 °C. Later, the cells were washed by ice-cold PBS three times and analysed using a FACSCalibur flow cytometer (Becton Dickinson). The analysis was performed by using a FlowJo X (Version 10.0.7) software.

### Lipid peroxidation assays

The lipid peroxidation product MDA concentration in GBM cell lysates was assessed using a Lipid Peroxidation MDA Assay Kit (S0131S, Beyotime, China) according to the manufacturer’s protocol. The total cell content was measured by a BCA kit (P0012, Beyotime). The absorbance of MDA and BCA was detected using a Multimode Plate Reader (PerkinElmer, Germany). Relative MDA concentration was calculated as MDA/BCA.

### Glutathione assays

GSH concentration in GBM cell lysates was measured using a Glutathione Assay Kit (S0053, Beyotime) according to the manufacturer’s instructions. The total cell content was measured by a BCA kit (Beyotime). The absorbance of GSH and BCA was detected using a Multimode Plate Reader (PerkinElmer). Relative GSH concentration was calculated as GSH/BCA.

### Transmission electron microscopy (TEM)

Cell samples of U87 were collected with a low speed centrifugation and fixed in electron fixation solution containing 2.5% glutaraldehyde (Servicebio). After the post-fixation of 1% osmium tetroxide and dehydration, the samples were embedded in Epon. The specimen sections were stained with uranyl acetate and examined on a transmission electron microscope (Hitachi HT7700, Tokyo, Japan).

### In vivo imaging of mouse xenografts

Injected U87 cells in mouse xenografts were labelled with luciferase. First, each tumor-bearing mouse was injected with 10 mg D-Luciferin potassium (ST196, Beyotime) via caudal vein. 20 min after the injection, mice were captured by an In Vivo Imaging System (IVIS) (Bruker Xtreme BI, Bruker, USA).

### Statistical analysis

The data in this research are from three independent experiments and presented as mean ± SD. Student’s t-test was used for data comparisons between two group. One-way analysis of variance (ANOVA) was used for data comparisons among three or more groups and the Student–Newman–Keuls (SNK) method was used for post-analysis. Chi-square test of independent four-table data was used in the microarray analysis. Statistical analyses were performed using SPSS (Version 19.0). A P value of less than 0.05 was considered as statistical significance.

## Results

### RND1 expression was downregulated in GBM and predicted a better prognosis of patients with GBM

**Fig. 1:**
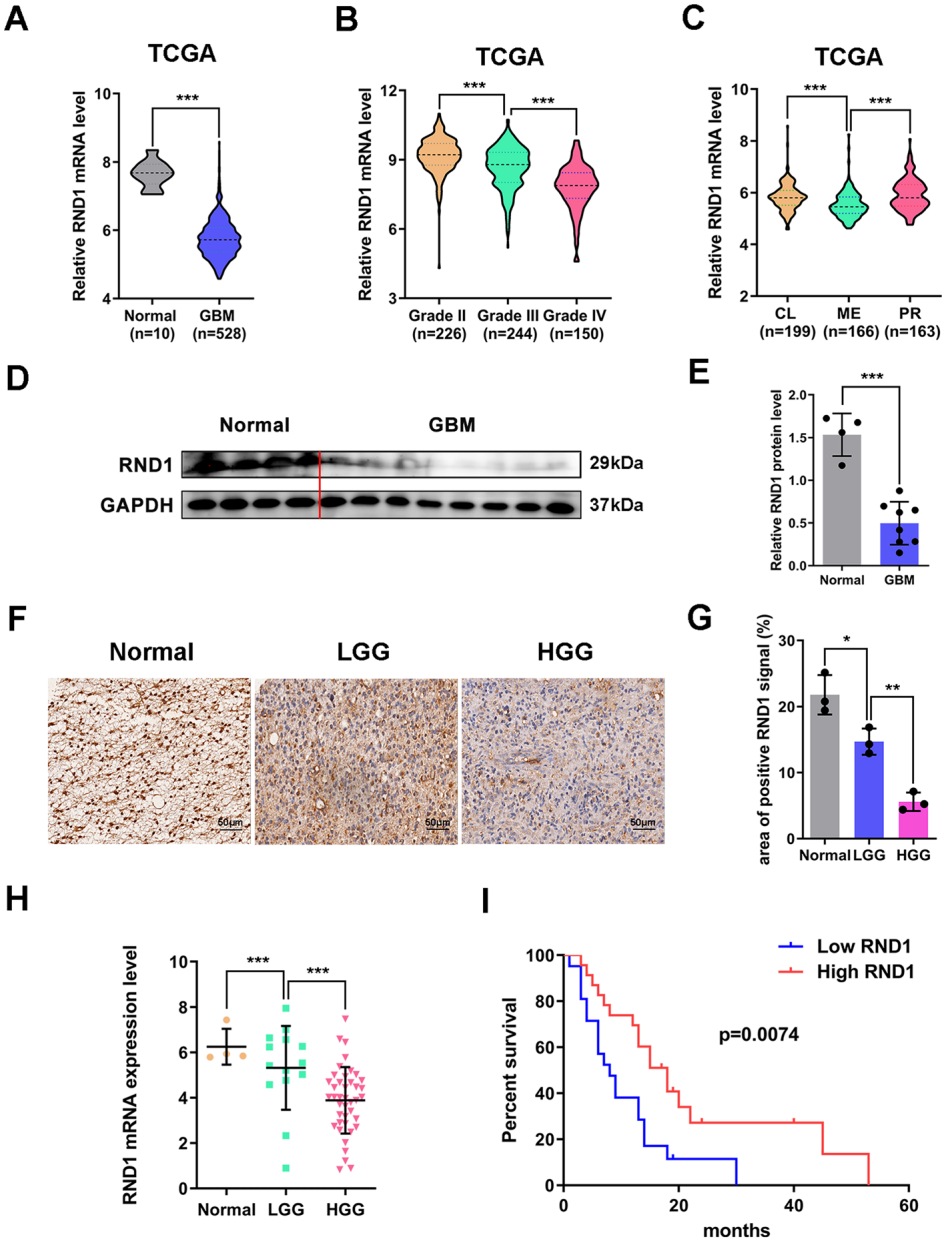
RND1 is downregulated and associates with prognosis in GBM. **A** Relative RND1 mRNA level in human normal brain (n = 10) and GBM tissues (n = 528) according to the TCGA dataset. **B** Relative RND1 mRNA level in grade II (n = 226), grade III (n = 244) and grade IV (n = 150) of glioma tissues based on TCGA dataset. **C** Relative RND1 mRNA expression in the CL (n = 199), ME (n = 166) and PR (n = 163) subtypes of GBM based on TCGA dataset. CL, classical; ME, mesenchymal; PR, proneural. **D** Western blot analysis of RND1 protein level in clinical human normal brain (n = 4) and GBM (n = 8) tissues. **E** Relative expression of RND1 protein in normal brain and GBM tissues was quantified. **F** IHC staining images for RND1 in clinical human normal brain, LGG and GBM tissues. Scale bars, 50 μm. LGG, low grade glioma. **G** Positive stained area of IHC staining for RND1 was quantified. **H** Relative RND1 mRNA expression of clinical human normal brain (n = 4), LGG (n = 14) and GBM (n = 41) tissues was quantified. **I** Kaplan–Meier survival analysis for clinical GBM patients with RND1 expression in tumor tissues. *P < 0.05; **P < 0.01; ***P < 0.001

Using Human Protein Atlas database, we investigated the expression levels of RND1 in various human normal tissues and tumor tissues. RND1 was predominantly found in human liver, lung, and brain tissues (Additional file 1: Fig. S1A), and it was down-regulated in various tumors (Additional file 1: Fig. S1D). We searched TCGA database to analyze RND1 expression in gliomas and found that RND1 mRNA level was down-regulated in GBM tissue compared to normal brain tissue (Fig. 1A) and that RND1 expression decreased with increasing glioma grade (Fig. 1B). The results were consistent with the analysis of clinical samples collected from humans (Fig. 1D–H). Relevant information on the tissue samples used above are listed in Additional file 8: Table S2.

Moreover, based on TCGA, we measured RND1 expression in subtypes of GBM, including classical (CL), mesenchymal (ME), and proneural (PR) GBM, where ME GBM, which shows the highest malignancy [18], had lower RND1 expression than CL and PR GBM (Fig. 1C). These findings showed that RND1 expression was down-regulated in GBM and negatively correlated with glioma malignancy.

RND1 expression in clinical GBM tissues was then further analyzed using RT-PCR assays and the prognosis of patients. Based on mRNA level of RND1, all samples were divided into high and low RND1 expression groups. In the subsequent Kaplan–Meier analysis, patients in the high RND1 expression group manifested a higher survival rate (Fig. 1I). Thus, RND1 predicted a better prognosis for patients with GBM and probably acted as a tumor suppressor gene in glioma. Relevant information on GBM samples used above are listed in Additional file 9: Table S3.

### RND1 inhibited glioma cell growth in a ferroptosis-dependent manner in vitro

**Fig. 2:**
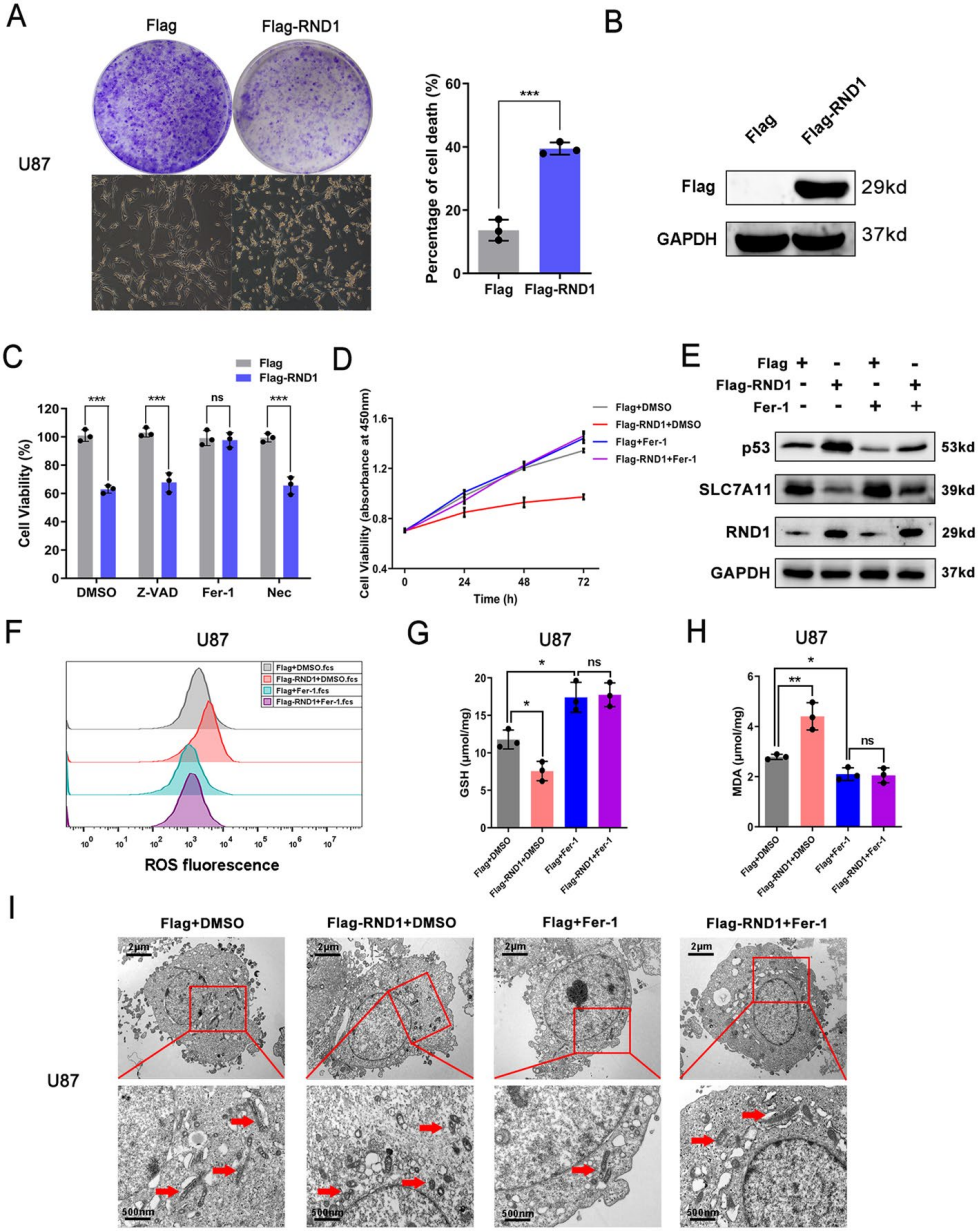
RND1 overexpression induced the ferroptosis of GBM in vitro. **A** Effects of RND1 overexpression on U87 cell growth according to the inverted microscope observation and colony formation assay. Ratio of inactive U87 cells under the inverted microscope and cell growth from colony formation assay were quantified. **B** The efficacy of Flag-RND1 plasmid was assessed by western blot analysis. **C** According to the cell viability assay, Fer-1 (10 µM) rescued the growth inhibition of U87 induced by RND1 overexpression after 48 h, while Z-VAD (20 µM) and Nec (20 µM) did not. Z-VAD, (Z-VAD (OMe)-FMK); Fer-1, Ferrostatin-1; Nec, Necrosulfonamide. **D** The cell viability assay showed that the duration of Fer-1 (10 µM) for 48 h or 72 h significantly inhibited the effect of RND1 overexpression in U87. **E** U87 cells were transfected with Flag or Flag-RND1 plasmids and treated with Fer-1 (10 μM). Western blot assays were used to measure the RND1, p53 and SLC7A11 protein levels. **F**–**H** The lipid ROS assay, glutathione assay and peroxidation assay also revealed a regulation of RND1 on the lipid ROS, GSH and MDA level of U87 cells, which was reversed by Fer-1 (10 µM, 48 h). ROS, reactive oxygen species; MDA, malondialdehyde; GSH, glutathione. **I** Transfected U87 cells were prepared for transmission electron microscopy observation. *P < 0.05; **P < 0.01; ***P < 0.001; ns, no significance

To confirm the function of RND1 in glioma, we designed RND1-overexpressing (Flag-RND1) and knockdown (shRND1) plasmids to conduct experiments in the p53 wild-type GBM cell lines U87 and A172. As a result of up-regulating RND1 expression, it was observed that GBM cells narrowed, lightened, and clumped together with stagnant cell growth as viewed under a microscope (Fig. 2A and Additional file 2: Fig. S2A). The cell growth was also examined using colony formation and cell viability assays, and the results revealed that overexpressing RND1 group exhibited significantly lower cell growth than the control group (Fig. 2A and Additional file 2: Fig. S2C). Several inhibitors were used to treat GBM cells to clarify RND1's role in regulating cell growth. A classic apoptosis inhibitor (Z-VAD-FMK) and a necrosis inhibitor (necrosulfonamide) could not reverse the effects of RND1 overexpression. However, a ferroptosis inhibitor (ferrostatin-1, Fer-1) reversed RND1’s cell-inhibiting effects (Fig. 2C). Therefore, we performed lipid ROS, lipid peroxidation, and glutathione assays to evaluate the changes in lipid oxidation produced during ferroptosis. Overexpression of RND1 significantly increased the level of lipid ROS and was rescued by Fer-1 (Fig. 2F). The malondialdehyde (MDA) is the decomposition product of membrane lipid peroxidation, and its levels reflect the degree of lipid peroxidation in the cell. RND1-overexpressing group accumulated MDA, which was eliminated by Fer-1 (Fig. 2H and Additional file 2: Fig. S2E). GSH is the primary source of sulfhydryl groups an important endogenous antioxidant, which plays an essential role in maintaining the proper redox state of the cell. Our results revealed that GSH of GBM cell lysates decreased in RND1 overexpression group, and this effect could also be rescued by Fer-1 (Fig. 2G and Additional file 2: Fig. S2D).

Furthermore, morphological changes in the microstructure of the cells were analyzed using transmission electron microscopy (TEM). The results indicated no ruptured nuclear membranes or pyroclastic bodies in cells from RND1 overexpression group, but typical ferroptotic characteristics, mitochondrial deformation, and mitochondrial crista thickening were observed. This phenomenon was reversed by Fer-1 (Fig. 2I). After knocking down RND1, consistent results were obtained (Additional file 3: Fig. S3).

### RND1 regulated the expression of SLC7A11 and p53 in GBM

**Fig. 3:**
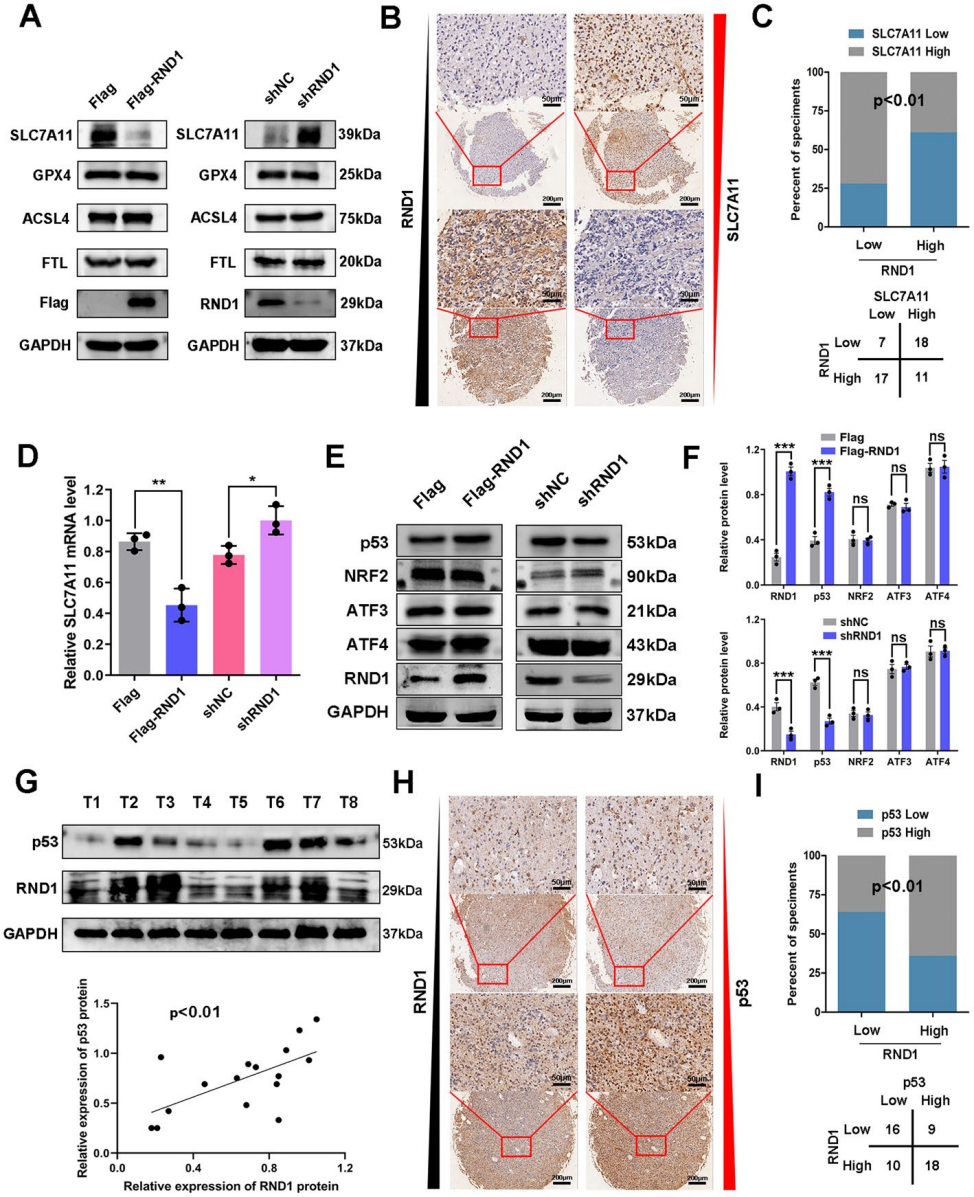
RND1 regulated the expression of SLC7A11 and p53 in GBM. **A** Influence of RND1 expression on the protein levels of SLC7A11, GPX4, ACSL4 and FTL in U87. **B** Representative images of IHC staining showed that RND1 expression inversely correlates with SLC7A11 in the same location of GBM tissues. Scale bars, 50 μm at high magnification and 200 μm at low magnification. **C** Microarray analysis of 53 GBM tissues showed the inverse correlations of IHC data for high or low RND1 expression relative to level of SLC7A11. **D** mRNA level of SLC7A11 when RND1 was overexpressed or knocked down in U87. **E**, **F** Influence of RND1 expression on the protein levels of p53, NRF2, ATF3 and ATF4 in U87. **G** Western blot analysis showed that RND1 expression positively correlates with p53 protein level in clinical GBM samples. **H** Representatives images of IHC staining showed that RND1 expression positively correlates with p53 in the same location of GBM tissues. Scale bars, 50 μm at high magnification and 200 μm at low magnification. **I** Microarray analysis of 53 GBM tissues showed the positive correlations of IHC data for high or low RND1 expression relative to level of p53. *P < 0.05; **P < 0.01; ***P < 0.001; ns, no significance

To determine the mechanism of RND1 in regulating ferroptosis in GBM cells, the expression of several core regulatory proteins of iron metabolism and lipid oxidation were evaluated, including SLC7A11, GPX4, ACSL4, and FTL [6, 19–21], when RND1 was overexpressed or knocked down (Fig. 3A and Additional file 4: Fig. S4A, B). It was observed that SLC7A11, a component of system x_c_^−^ that maintains the stability of lipid oxidation in cells, was negatively regulated by RND1 in vitro (Fig. 3 and Additional file 4: Fig. S4). Similarly, employing analysis of tissue microarrays containing 53 GBM samples, it was observed that the expression level of SLC7A11 was negatively correlated with RND1 (Fig. 3B, C). These results indicated a negative regulatory effect of RND1 on SLC7A11.

To verify the mechanism by which RND1 regulates SLC7A11, we detected several upstream regulatory signals of SLC7A11, including ATF3, ATF4, p53, and NRF2 [6, 22–24]. Western blot assays disclosed that the protein levels of ATF3, ATF4, and NRF2 demonstrated no significant difference when RND1 was overexpressed or knocked down in GBM cells (Fig. 3E and Additional file 4: Fig. S4C, D). However, we detected a significant change in p53 protein levels, and p53 was positively regulated by RND1. Furthermore, the protein expression of RND1 and p53 analysis in 16 GBM samples revealed that the expression level of p53 was related to RND1 (Fig. 3G). The analysis of tissue microarrays analysis also confirmed that p53 expression was positively correlated with RND1 in GBM samples (Fig. 3H, I). Based on these results, RND1 up-regulates p53 expression and down-regulates SLC7A11 expression in GBM.

### RND1 inhibited the progression of glioma in vivo

**Fig. 4:**
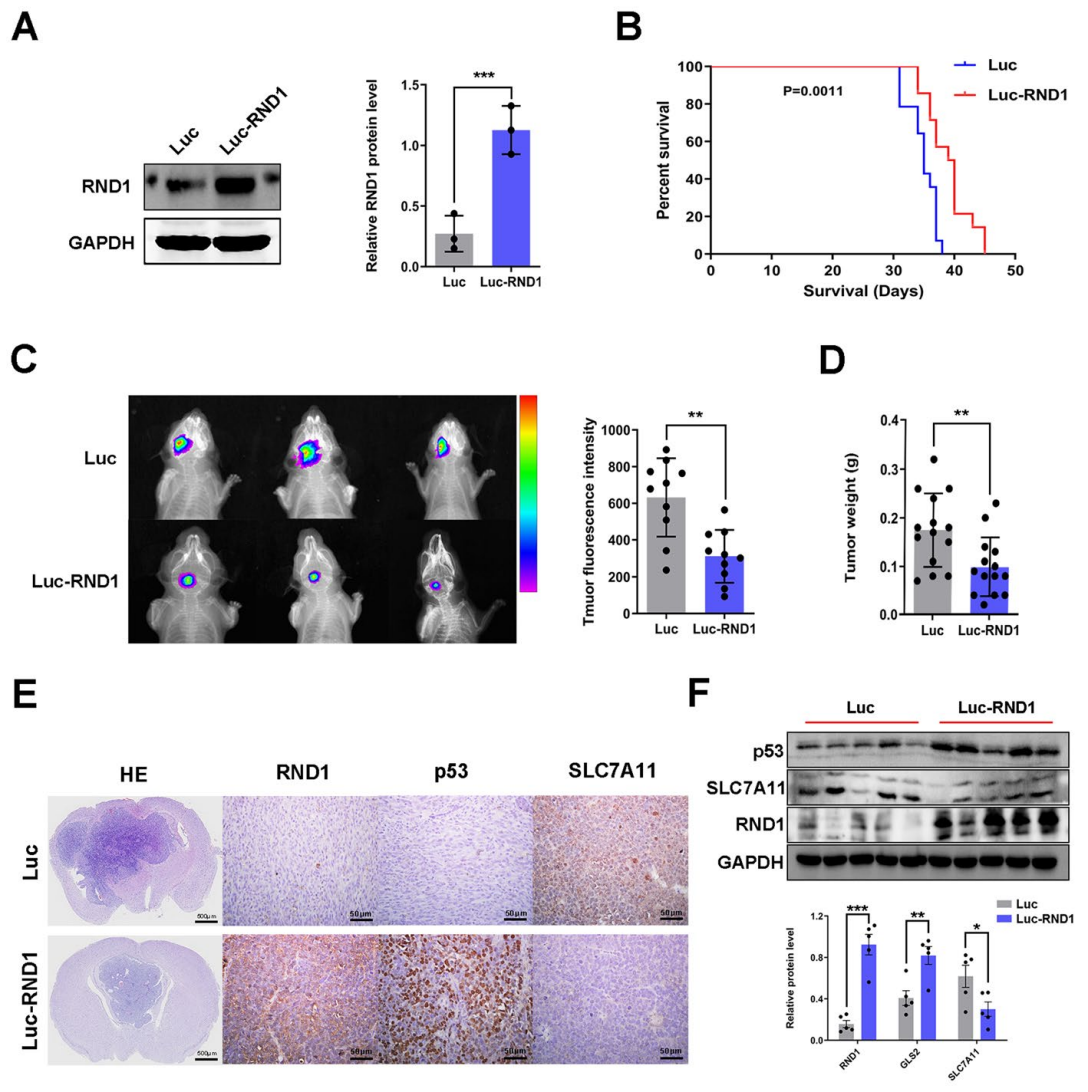
RND1 suppressed the growth of GBM in vivo. **A** The establishment efficacy of stable cell line with luciferase was assessed by western blot analysis. **B** Mouse survival was shown by Kaplan–Meier curves. U87-Luc groups, n = 14; U87-Luc-RND1 groups, n = 14. P value was calculated using the log-rank test. P = 0.0011. **C** RND1 suppressed the growth of GBM in the intracranial xenograft model. Luciferase fluorescence of mouse xenografts were detected by an IVIS at the 21st day after tumor injection. Luciferase activities were quantified. **D** The weight of tumors were analysed (Luc/Luc-RND1 groups—PBS groups). **E** Representative HE staining images of orthotopic tumor sections confirmed the tumor suppression in Luc-RND1 groups. **F** Protein levels of p53, SLC7A11, and RND1 in mouse tumor were also evaluated by western blot and quantified. IHC staining and western blot revealed that RND1 regulated the expression of p53 and SLC7A11 in vivo. Luc, Luciferase. *P < 0.05; **P < 0.01; ***P < 0.001

To confirm the function of RND1 in glioma, an in vivo intracranial xenograft model of GBM was developed. Using lentivirus, two stable U87 cell lines (U87-Luc and U87-Luc-RND1) were established, and Western blot confirmed their efficiency (Fig. 4A). An IVIS was used to evaluate mouse xenografts labeled with luciferase to generate firefly fluorescence. From the fluorescence, it was observed that tumor growth in Luc-RND1 group was significantly inhibited compared to that of the control group (Fig. 4C). Survival analysis revealed that the survival of the nude mice in luciferase-RND1 group was also higher than that in the control group (Fig. 4B). By comparing the relative size and growth rate of tumors from both groups, it was observed that tumor growth of the nude mice was significantly inhibited in Luc-RND1 group compared with the control group (Fig. 4D and Additional file 5: Fig. S5B), which was later confirmed by H&E staining of mouse brain sections (Fig. 4E). Furthermore, we detected the expression of RND1, p53, and SLC7A11 through immunohistochemical staining and western blot and discovered that p53 expression was up-regulated, while SLC7A11 expression was down-regulated in brain tissues from Luc-RND1 group (Fig. 4E, F). These results demonstrated that RND1 induced ferroptosis and inhibited glioma progression in vivo.

### RND1 interacted with p53 and inhibited the degradation of p53 protein.

**Fig. 5:**
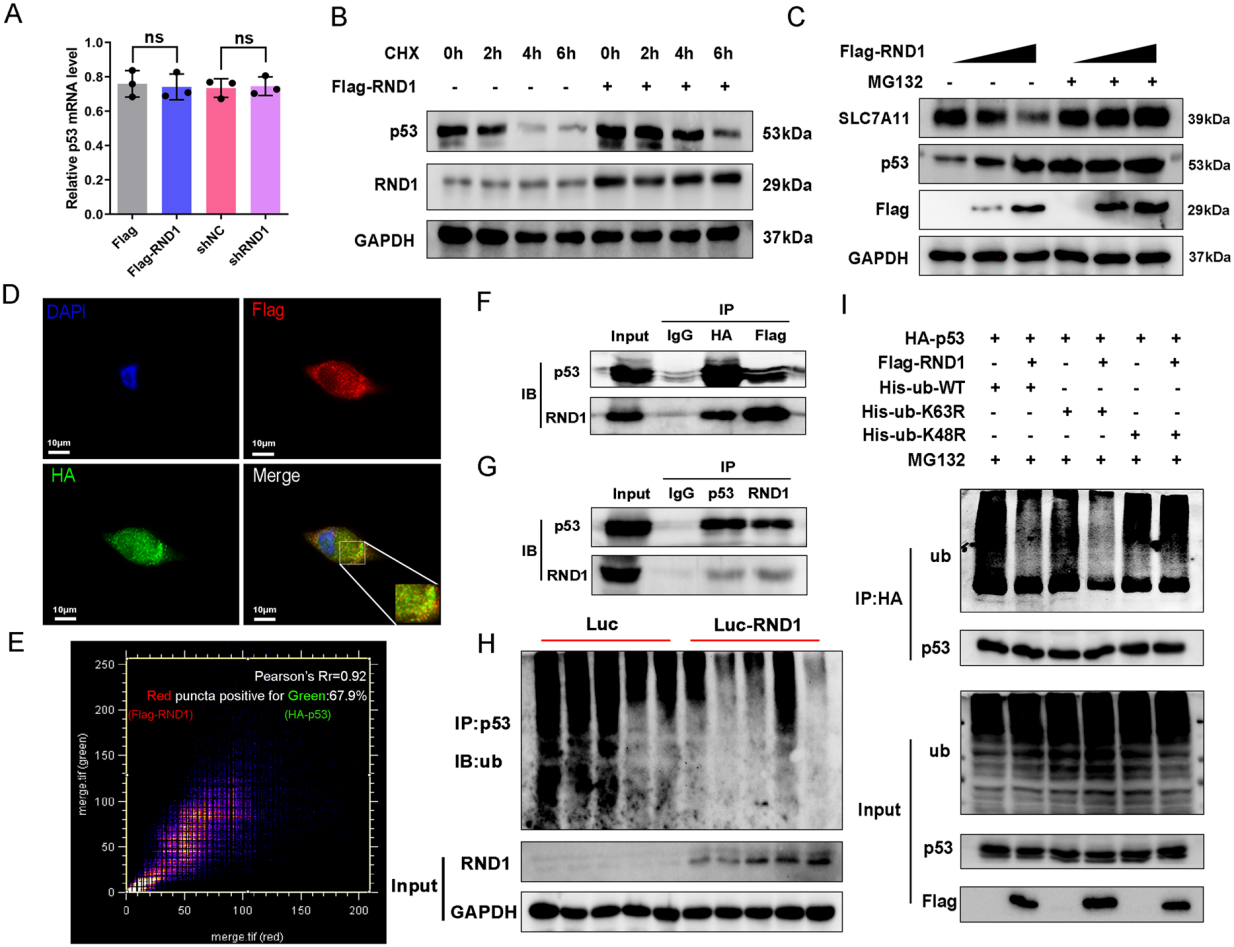
RND1 physically interacted with p53 and inhibited p53 protein degradation. **A** The transcript levels of p53 remained unchanged under overexpression and down-regulation of RND1 circumstances. **B** Overexpression of RND1 promoted the stability of p53. After the cells were treated with CHX (50 μM) for different time periods (0, 2, 4, 6 h), p53 protein levels were analyzed by western blot. CHX, cycloheximide. **C** From western blot, proteasome inhibitor MG132 (10 μM, 6 h) blocked the regulation of p53 protein by RND1. **D** Immunofluorescent analysis of the co-localization of exogenous RND1 (labelled with Flag) and p53 (labelled with HA) proteins in U87 cells. Scale bar, 10 μm. **E** The colocalization between Flag-RND1 and HA-p53 has been quantified using the Colocalization Finder of Image J. Pearson’s Rr = 0.92; Overlap R = 0.93. **F** Co-IP pull-downs were conducted to investigate the association between RND1 and p53 at the exogenous level. 293T cells were co-transfected with Flag-RND1 and HA-p53 plasmids, which were later subjected to IP using anti-Flag, anti-HA antibodies, followed by immunoblotting with anti-RND1 and anti-p53 antibodies. **G** Co-IP pull-downs were conducted to investigate the association between RND1 and p53 at the endogenous level. **H** RND1 inhibited p53 ubiquitination in vivo. Proteins used for IP in vivo were extracted from tumor tissues of mouse xenografts. The loading proteins were precipitated by p53 antibody and immunoblotted with the anti-ub antibody. **I** RND1 inhibited the K48-linked polyubiquitin of p53 protein and stabilized p53. After 293T cells were transfected with HA-p53 and His-ub-WT, His-ub-K48R or His-ub-K63R, the cell lysates were subjected to IP using an anti-HA antibody, followed by immunoblotting with the anti-ub antibody. ns, no significance

Despite a significant up-regulation of p53 protein expression when RND1 expression was elevated, p53 mRNA levels remained unchanged. When RND1 was knocked down, p53 protein expression rather than mRNA expression decreased (Figs. 3E and 5A), suggesting p53 is post-transcriptionally regulated by RND1. Using cycloheximide (CHX) to block translation in U87 cells, it was observed that p53 gradually degraded with the extension of CHX processing time, whereas p53 degradation was delayed in RND1-overexpressing group (Fig. 5B). Moreover, the proteasome inhibitor MG132 reversed the regulatory effect of RND1 on p53 protein levels (Fig. 5C).

To elucidate the intracellular mechanism by which RND1 mediated p53, Flag-RND1 and HA-p53 were transferred into U87 cells. Immunofluorescence staining revealed that the subcellular localization of exogenous RND1 (labeled with Flag-tag) and p53 (labeled with HA-tag) mostly overlapped (Fig. 5D, E). Co-IP assays detected p53 protein in the precipitates pulled down by Flag and RND1 protein in the precipitates pulled down by HA, indicating that RND1 associates with p53 (Fig. 5F). Co-IP between endogenously expressed RND1 and p53 proteins was also analysed (Fig. 5G).

The p53 protein is fundamentally regulated by the ubiquitin–proteasome system (UPS), and ubiquitinating or deubiquitinating enzymes regulate p53 protein degradation. Additionally, we investigated whether the binding of RND1 to p53 affected the level of ubiquitination of p53. As indicated in Fig. 5, we measured the ub level in the precipitates pulled down by HA-tag, labeled p53, and the results indicated that RND1 overexpression decreased p53 protein ubiquitination (Fig. 5I). The function of the modification of polyubiquitination of lysine 48 (K48) is to form the ub-proteasome system to degrade substrate protein [25], and the mutation of K48 to arginine renders RND1 unable to deubiquitinate p53, implying that RND1 suppressed K48-linked polyubiquitin of p53 protein and stabilized p53 (Fig. 5I). To investigate whether RND1 inhibited p53 ubiquitination in vitro, the proteins from tumor tissues of mouse xenografts were extracted. The loading proteins were precipitated by a p53 antibody and immunoblotted with an anti-ub antibody. A significant reduction of p53 ubiquitination in tumor tissues of the Luc-RND1 group was observed (Fig. 5H).

### RND1 induced ferroptosis through the p53-SLC7A11 signaling pathway

**Fig. 6:**
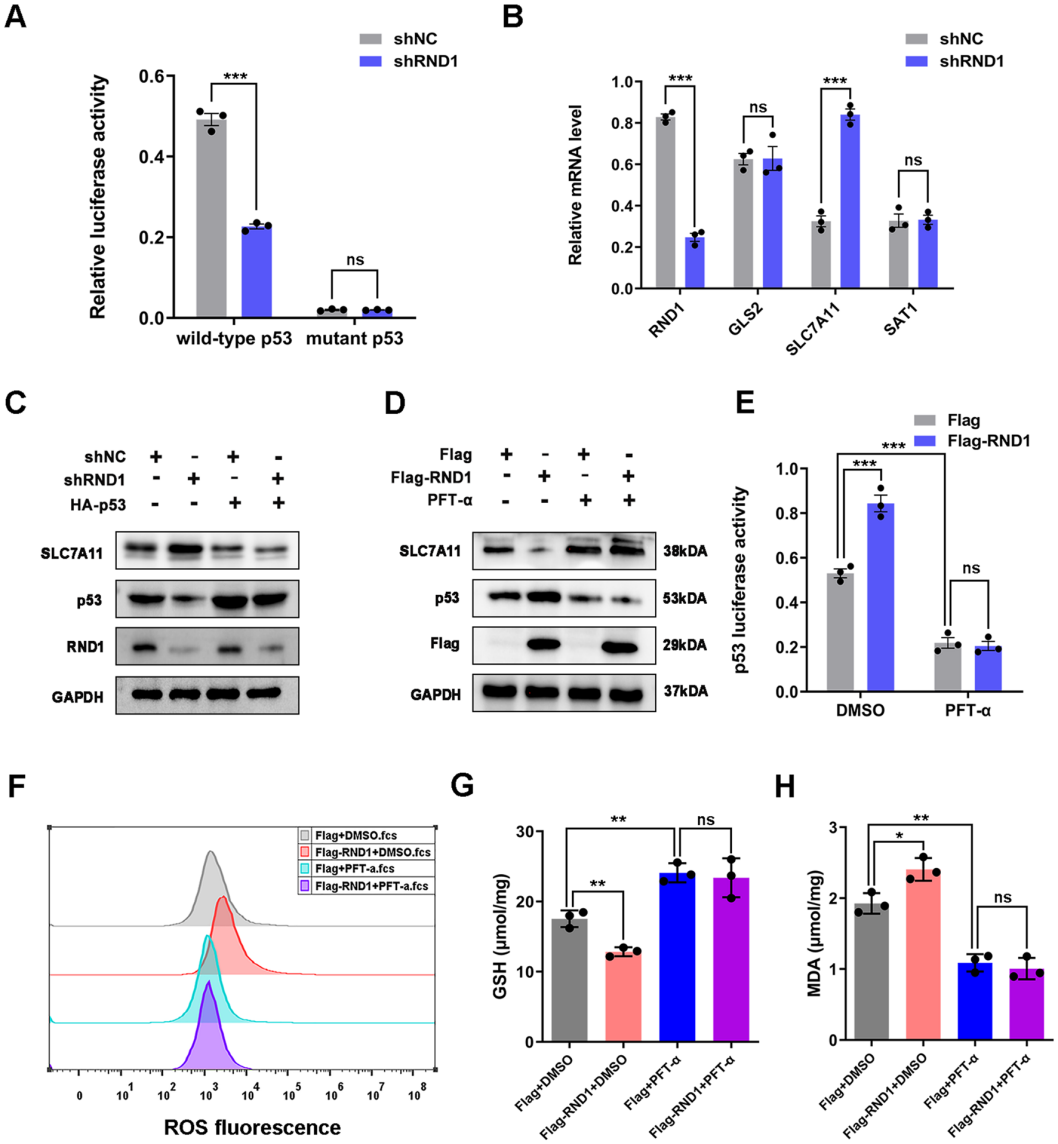
RND1 induced the ferroptosis via the p53-SLC7A11 signaling in GBM. **A** Knockdown of RND1 significantly decreased the p53-driven luciferase activity in U87 cells by dual luciferase reporter assays. **B** Knockdown of RND1 increased the mRNA level of SLC7A11. **C** HA-p53 reversed the effects of RND1 knocked down on p53 and SLC7A11 protein levels in U87 cells. **D** PFT-α (an inhibitor of p53) reversed the effects of RND1 overexpression on p53 and SLC7A11 protein levels in U87 cells. **E** According to the dual luciferase reporter assays, PFT-α reversed the activity of p53 signaling when RND1 was overexpressed. **F**–**H** PFT-α reversed the effects of RND1 overexpression on the ROS, GSH level and MDA level in U87 cells. *P < 0.05; **P < 0.01; ***P < 0.001; ns, no significance

As a next step, dual-luciferase reporter assays were used to determine whether RND1 inhibits the activity of the p53 signaling pathway (Fig. 6A and Additional file 6: Fig. S6A). To identify whether RND1 activation of p53 signaling induces ferroptosis by regulating SLC7A11, the expression of several molecules downstream of the p53 signaling pathway involved in ferroptosis was measured, including GLS2, SLC7A11, and SAT1 [26]. PCR assays showed that silencing RND1 did not alter GLS2 and SAT1 expressions but increased SLC7A11 mRNA levels (Fig. 6B). When RND1 was knocked down, we detected less p53 and more SLC7A11, and transfection of HA-p53 reversed this effect (Fig. 6C). Using PFT-α, an inhibitor of p53 gene transcription, reversed the activity of p53 signaling and the expression of SLC7A11 when RND1 was overexpressed, suggesting a p53-SLC7A11 signal mediated by RND1 (Fig. 6D, E). Moreover, PFT-α affected RND1’s control of the intracellular concentration of GSH (Fig. 6F–H), whose synthesis is affected by system x_c_^−^ and cysteine uptake, thus influencing the level of MDA in GBM cells. As a result, recovery of p53 function in combination with HA-p53 effectively suppressed ROS-induced stress in GBM cells (Additional file 6: Fig. S6).

### RND1 promoted the anti-GBM activity of erastin.

**Fig. 7:**
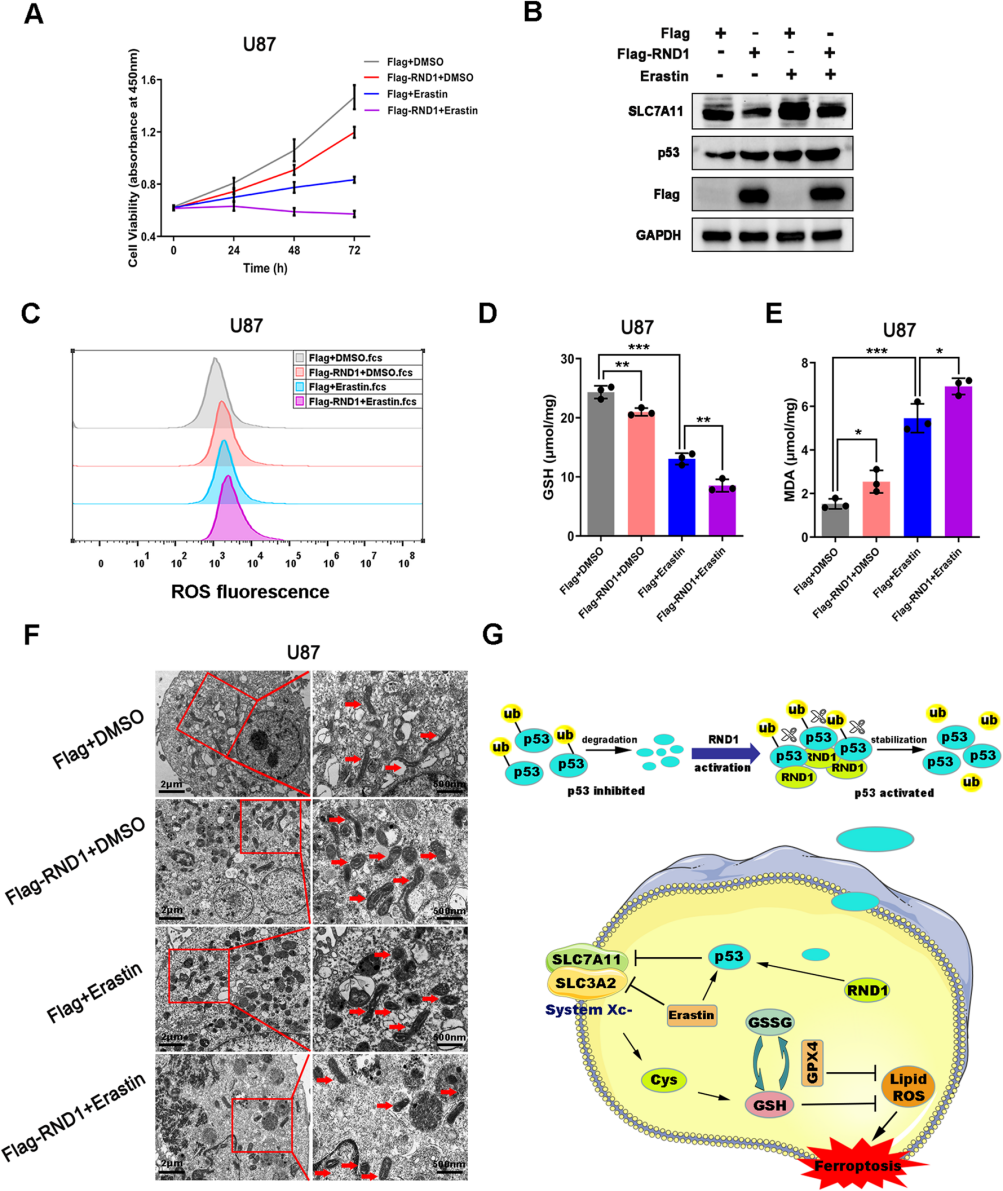
RND1 promoted the anti-GBM activity of Erastin in vitro. **A** RND1 overexpression combined with Erastin significantly suppressed the growth of U87 cells. Cell viability was detected using CCK-8. **B** U87 cells were transfected with Flag or Flag-RND1 plasmids and treated with Erastin (10 μM). Western blot analysis was used to determine the levels of SLC7A11 and p53. **C**–**E** RND1 overexpression combined with Erastin significantly decreased the concentration of GSH and increased the level of ROS and MDA in U87 cells. **F** Transfected U87 cells were treated with Erastin (10 μM) and prepared for transmission electron microscopy observation. **G** Mechanistic model for RND1 regulation of cell ferroptosis in GBM. RND1 directly interacted with p53, leading to the de-ubiquitination and reduced degradation of p53 protein. Further, it results in higher activity of p53-SLC7A11 signaling pathway, thus enhancing the ferroptosis of GBM. *P < 0.05; **P < 0.01; ***P < 0.001

Erastin, an essential inducer of ferroptosis, could inhibit the function of system x_c_^−^ and activate p53 in ferroptosis [27], which was like the effect of RND1 on the regulation of ROS-dependent ferroptosis. To evaluate whether erastin-induced ferroptosis could be affected by RND1, U87 cells were treated with DMEM containing 10 μM erastin and changed RND1 expression. Western blot results showed that RND1 and erastin increased p53 protein levels. Interestingly, although p53 expression was up-regulated, SLC7A11 protein remained at a high level after adding erastin and remained down-regulated when RND1 was overexpressed (Fig. 7B). Phenotypically, the overexpression of RND1 combined with erastin significantly reduced the levels of intracellular GSH and increased MDA concentration (Fig. 7D–F), which ultimately induced ferroptosis and inhibited the growth of U87 cells (Fig. 7A). More deformed mitochondria with thickened mitochondrial cristae were found through TEM observation in RND1 combined with the erastin group (Fig. 7C). Transfection of U87 cells with Flag-SLC7A11 allowed us to investigate whether SLC7A11 was involved in ferroptosis regulation by RND1. Cys and GSH concentrations were rescued by Flag-SLC7A11 to a certain extent, reflecting a particular function of SLC7A11 in regulating ferroptosis by RND1 upon erastin stimulation (Additional file 6: Fig. S6D, E and F). Based on these results, we have demonstrated that RND1 enhanced the antitumor activity of erastin in U87 cells through ferroptosis.

## Discussion

This study discovered the interplay effects of RND1 and p53 proteins where RND1 is reported to be a positive regulator of p53 protein and induces ferroptosis of GBM cells. The up-regulation of RND1 expression inhibited the degradation of p53 protein via de-ubiquitination and activated p53-SLC7A11 axis in GBM. The analysis of clinical GBM samples has also confirmed that RND1 serves as a prognostic indicator. These findings may provide a novel therapeutic target for treating GBM.

Uncontrolled programmed cell death (PCD) is a typical characteristic of GBM and a significant reason for resistance to treatment and poor prognosis of glioma patients [28]. Ferroptosis is another type of PCD requiring iron metabolism and unique morphological and biological characteristics: deformed and atrophied mitochondria with increased membrane density and reduced mitochondrial cristae [29]. The regulatory mode of ferroptosis is also different from traditional PCD, which is mainly manifested in cystine/glutamate antitransport system (system x_c_^−^)—glutathione peroxidase 4 (GPX4) axis inhibition and reactive oxygen species (ROS) production by unmetabolized peroxide, leading to cell membrane lipid peroxidation and cell death [30]. The ferroptosis inducer erastin has been shown to increase the sensitivity of GBM cells to temozolomide chemotherapy [31, 32]. Moreover, signaling pathways such as ATF4, NOX4 and NCOA4 were shown to be involved in regulating the ferroptosis of GBM cells [33–35]. Because current research on the mechanism and potential targets of ferroptosis in glioma is insufficient, this issue requires further investigation.

RND1 is a small GTPase that belongs to RND subfamily. RND1 protein's function depends on epigenetic, transcriptional, post-transcriptional, and post-translational mechanisms rather than GDP/GTP exchange [12]. Under normal circumstances, RND1 is also involved in biological angiogenesis, neural connections and embryonic development [12]. Researchers investigated the tumor suppressor effect of RND1 in several tumors, and the influence of RND1 on EMT was discovered. Notably, a study on glioma stem-like cells (GSCs) demonstrated that RND1 regulated the invasion of GSCs in the periventricular zone [16]. Because research on RND1 in cancer mainly focuses on the cell phenotype of EMT, we studied RND1’s effect on lipid oxidation in GBM cells and its regulation mechanism in ferroptosis.

We first analyzed RND1 expression in glioma samples obtained from public databases and collected clinical samples. TCGA database analysis revealed that RND1 expression levels were significantly lower in GBM than in normal brain tissue and that RND1 expression was negatively correlated with glioma grade. GBM exhibits considerable molecular heterogeneity and transcriptional heterogeneity. According to transcriptional cell status and gene alteration pattern, GBM was divided into four subtypes: classic (CL), neuronal type (NE), pre-neuronal type (PR), and mesenchymal (ME), where ME type tends to show the highest malignancy in GBM [18]. Hence, we compared RND1 expression in GBM subtypes and found that RND1 was markedly decreased in ME type compared with PR and CL types. Additionally, in the analysis of GBM samples collected clinically, RND1 was positively correlated with patient prognosis, indicating that RND1 is a potential prognostic predictor and therapeutic target for GBM.

This study revealed a novel function of RND1-induced ferroptosis in GBM. RND1 markedly inhibited glioma growth in vitro, as evaluated by cell viability assays and colony formation assays. Furthermore, we observed a phenomenon of cell death in which GBM cells narrowed, lightened, and clumped under microscope. To study the intrinsic effect of RND1 on the inhibition of glioma growth, we used Z-VAD-FMK, necrosulfonamide, and Ferrostatin-1 to reverse apoptosis, necrosis, and ferroptosis in glioma cells, respectively. The Fer-1 reversed tumor suppression induced by RND1 overexpression compared to Z-VAD-FMK and necrosulfonamide, suggesting that RND1 tumor inhibition of RND1 was not caused by apoptosis or necrosis, but by ferroptosis instead. Cancer cells rely on their robust antioxidant capacity to escape ferroptosis [36], while GSH depletion in cancer cells can inactivate GPX and accumulate lipid peroxidation [37]. RND1 inhibited the level of GSH and induced lip ROS in glioma cells. There is a correlation between RND1 and cell migration in GBM stem cells [16], and several studies have shown that the sensitivity of tumor cells to ferroptosis depends on their EMT status [38, 39]. Therefore, in the future, we must examine whether EMT caused by RND1 might improve the sensitivity of gliomas to ferroptosis.

In particular, we found that RND1-mediated ferroptosis was critically regulated by p53-SLC7A11 signaling in glioma. SLC7A11 expression, an essential protein in the ferroptosis program, representing the component of system x_c_^−^ that mediates the uptake of cystine and maintains lipid oxidation stability [22], was downregulated by RND1. Previous studies have revealed that SLC7A11 can be regulated by several genes, such as p53, NRF2, ATF3, and ATF4 [40]. We detected these necessary upstream signals in clinical GBM samples in vitro and found that RND1 significantly positively regulated p53. Moreover, RND1 promoted the activity of the p53 signaling pathway in GBM cells.

The p53 signaling pathway is a double-edged sword that can either have beneficial effects or be inhibitory in ferroptosis. First, p53 can inhibit SLC7A11, leading to L-Cysteine (Cys) deprivation [29]. p53 can also increase GSH breakdown by regulating GLS2 expression [41]. Furthermore, p53 can regulate PUFA synthase by acting on SAT1, increasing lipid peroxide synthesis and cell susceptibility to ferroptosis [42]. Alternatively, Cys deprivation activates the p53-p21 signaling pathway, increasing GSH levels and inhibiting GPX4 function [43]. Additionally, p53 can inhibit DPP4-NOX1 signaling pathway-dependent ROS production, ultimately inhibiting cellular susceptibility to ferroptosis [44]. To determine the specific mechanism by which RND1 affects the p53 signaling pathway, we detected the levels of SLC7A11, GLS2, and SAT1, which are vital molecules downstream of p53 in ferroptosis. From qPCR analysis, only mRNA of SLC7A11 was affected by RND1 knockdown, demonstrating that RND1 induces ferroptosis via p53-SLC7A11 pathway.

Another interesting aspect of this project was that RND1 increased the p53 protein expression without affecting p53 mRNA levels, suggesting that RND1 regulates p53 post-transcriptionally, as confirmed via employing confocal microscopic imaging and co-immunoprecipitation techniques, which revealed that RND1 interacts with p53. Ubiquitination plays a vital role in p53 protein regulation. The ubiquitin ligases of E3 represented by Mdm2 are the main negative regulators of p53, which cause degradation of p53 protein by promoting p53 polyubiquitination [7]. In contrast, the ubiquitination level of p53 protein can be removed by deubiquitinase (DUB) [9]. The regulation of de-ubiquitination of p53 protein has been a hotspot and has contributed to our understanding of different tumors [9, 45, 46]. In the ubiquitination assay, RND1 decreased the ubiquitination level of p53 protein, and using MG132 blocked p53 regulation by RND1. Polyubiquitin chains are formed by seven lysine sites of ubiquitin, represented by K48 and K63 [25]. The function of K48 polyubiquitination modification is to form a ub-proteasome system to degrade substrate protein [25], while K63 modification functions in DNA damage repair, translation, and signal pathway regulation [47]. To further explore the site of p53 de-ubiquitination affected by RND1, we constructed the ub-K48R (mutation of lysine 48 to arginine) and ub-K63R (mutation of lysine 63 to arginine) plasmids to conduct the ubiquitination assays. A mutation of lysine 48 to arginine renders RND1 incapable of deubiquitinating p53, implying that binding RND1 to p53 inhibited polyubiquitination of the p53 protein, stabilizing it.

However, the specific mechanism of p53 de-ubiquitination by RND1 was still not clarified in our study. Although it was indicated that RND1 interacts with p53, it is yet unclear whether this interaction is directly bound, cross-linked, or formed as a protein complex. On the one hand, RND1 may directly act as a DUB of p53 protein and stabilize p53, while on the other hand, RND1 may indirectly lead to p53 de-ubiquitination by interacting with E3 ligases or DUBs, considering p53 is usually ubiquitinated by E3 ubiquitin ligases represented by Mdm2 or stabilized by other DUBs such as HAUSP, OTUD5 and USP49 [45, 46, 48]. Furthermore, recent studies have revealed a new layer of regulation for Rho GTPases, indicating that several members of Rho family of small GTPases, including RhoA, Rac1, and RhoBTB, as well as Ras family member Rap1B, are also regulated by the ubiquitin–proteasome pathway [49, 50]. As a small GTPase member of Rho family, RND1 may also be regulated by ubiquitination. It remains to be determined whether RND1 ubiquitination affected p53. Overall, our research on RND1 suggests a molecule that affects p53 de-ubiquitination and deserves further investigation.

Erastin is a ferroptosis inducer that has been experimentally used in a variety of tumors, including gliomas[27]. Previous research revealed that Erastin sensitized glioblastoma cells to temozolomide by restraining system x_c_^−^ [32]. Similar results were also obtained in this study where RND1 activated the p53 and induced ferroptosis. The activation of p53 signaling pathway after ROS accumulation was universally observed [6, 51]. However, the inhibitory effect of erastin on system x_c_^−^ might lead to compensatory transcriptional upregulation of SLC7A11, which was also observed by Lo and Dixon [29, 52]. Although expression levels of SLC7A11 were up-regulated, the Cys assay showed that the intracellular Cys level decreased, and the level of GSH, which was synthesized from Cys, also declined, reflecting the decreased transport activity of system xc-. Moreover, Flag-SLC7A11 partially restored the Cys and GSH concentrations, suggesting that SLC7A11 plays a role in regulating ferroptosis by RND1 upon erastin stimulation. As a result, RND1 promoted erastin's anti-GBM activity via SLC7A11-mediated ferroptosis, which may have clinical significance in GBM.

## Conclusion

In summary, our study demonstrated that high RND1 expression level was associated with a lower malignancy in gliomas and a favourable prognosis for patients with GBM. RND1 bound to p53 and deubiquitinated the p53 protein, thus inhibiting the degradation of the p53 protein (Fig. 7G). RND1 facilitated ferroptosis in GBM by influencing p53-SLC7A11 signaling in vitro and in vivo. Moreover, RND1 enhanced the anti-GBM activity of erastin. Hence, RND1 could be a prognostic predictor and a potential therapeutic target for GBM.

## Supplementary Information

**Additional file 1: Figure S1.** The expression of RND1 in normal tissues and different tumors. (A) The relative mRNA level of RND1 in normal human tissues. The expression profiles were downloaded from the HPA database. (B) The specificity of RND1 antibody was verified by western blot analysis. The anti-RND1 antibody did not show the non-specific reactions with other RND members, such as RND2 and RND3. (C) The expression of RND1 in four human GBM cell lines (U87, A172, U118 and U251) was analyzed by western blot assays. RND1 was relatively highly expressed in U87 and A172. (D) The expression of RND1 in different tumors according to TCGA database.

**Additional file 2: Figure S2.** RND1 overexpression induced the ferroptosis in A172. (A) RND1 suppressed the growth of A172 and reversed by Fer-1 according to the inverted microscope observation and colony formation assay. (B) A172 cells were transfected with Flag or Flag-RND1 plasmids and treated with Fer-1 (10 μM). (C) Cell viability assay showed that the duration of Fer-1 (10 µM) significantly inhibited the effect of RND1 overexpression in A172. (D-E) The glutathione assay and peroxidation assay revealed a regulation of RND1 on the GSH and MDA level of A172 cells, which was reversed by Fer-1 (10 µM, 48 h). *, *P* < 0.05; **, *P* < 0.01; ***, *P* < 0.001; ns, no significance.

**Additional file 3: Figure S3.** RND1 knockdown promoted cell growth and inhibited ferroptosis in U87. (A-B) Effects of RND1 knockdown on U87 cell growth according to the inverted microscope observation. The inactive cells were quantified. (C) The cell viability assay showed that RND1 knockdown promoted cell growth. (D-E) The glutathione assay and peroxidation assay revealed a regulation of RND1 on GSH and MDA level of U87 cells. (F) Transfected U87 cells were prepared for transmission electron microscopy observation. *, *P* < 0.05; **, *P* < 0.01.

**Additional file 4: Figure S4.** RND1 downregulated SLC7A11 and upregulated p53 in vitro. (A) The protein levels of SLC7A11, GPX4, ACSL4 and FTL were analyzed when RND1 was overexpressed in U87. (B) The protein levels of SLC7A11, GPX4, ACSL4 and FTL were analyzed when RND1 was knocked down in U87. (C) The protein levels of p53, NRF2, ATF3 and ATF4 were analyzed when RND1 was overexpressed in U87. (D) The protein levels of p53, NRF2, ATF3 and ATF4 were analyzed when RND1 was knocked down in U87. (E–F) According to western blot assays, RND1 downregulated SLC7A11 and upregulated p53 in A172. (G-H) The protein levels of SLC7A11 and p53 were analyzed when RND1 was overexpressed or knocked down in A172.

**Additional file 5: Figure S5.** RND1 suppressed the growth of GBM in vivo. (A) Representative images of mouse brains in intracranial xenograft models. (B) The growth of tumor in xenograft models was analyzed as tumor weight /survival.

**Additional file 6: Figure S6.** p53 acted a key role in RND1-induced ferroptosis in GBM. (A) Knockdown of RND1 significantly decreased the p53-driven luciferase activity in A172, which was rescued by HA-p53. The luciferase activity was calculated as Firefly luciferase/Renilla luciferase. (B) Knockdown of RND1 significantly inhibited the MDA level in A172, which was reversed by HA-p53. (C) Trough the observation of transmission electron microscopy, knocked down of RND1 suppressed the ferroptosis of A172, which was reversed by HA-p53. (D) RT-PCR showed that SLC7A11 was upregulated with Erastin stimulation and downregulated in the RND1 overexpressing cells. (E–F) Cys and GSH assays showed function of SLC7A11 in the regulation of ferroptosis by RND1 upon Erastin stimulation. *, *P* < 0.05; **, *P* < 0.01; ***, *P* < 0.001; ns, no significance.

**Additional file 7: **Sequences of genes, plasmids and primers.

**Additional file 8: **Clinical information for patients diagnosed with glioma.

**Additional file 9: **Clinical information for patients diagnosed with glioblastoma.

## Data Availability

The data used to support the findings of this study are available from the corresponding author upon request.
