# Supplementary figures and images for: Rho family GTPase 1 (RND1), a novel regulator of p53, enhances ferroptosis in glioblastoma
Source: Cell Biosci. 2022 May 3;12:53. doi: 10.1186/s13578-022-00791-w (PMC9066768; doi:10.1186/s13578-022-00791-w)

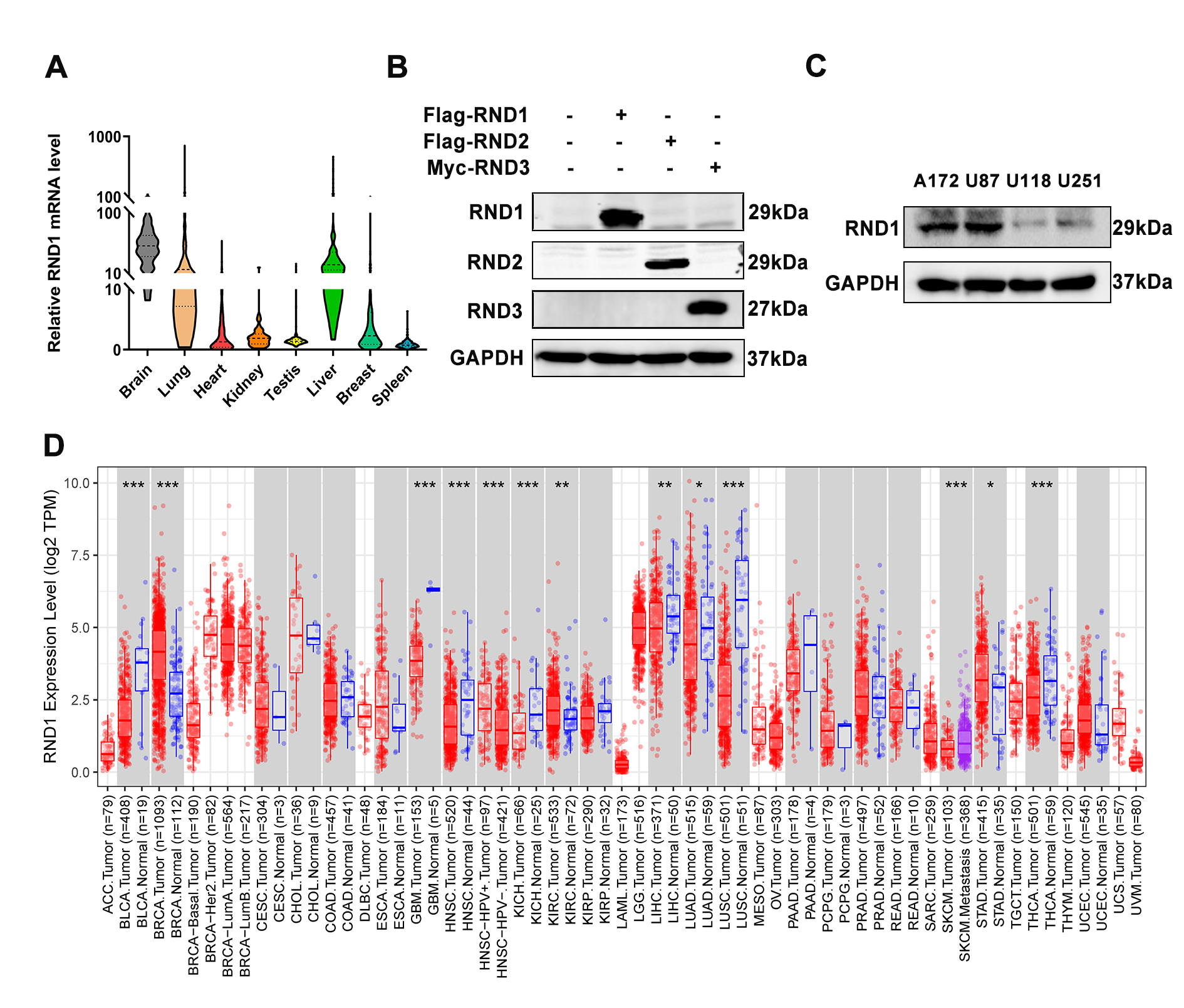

Supplement: Supplementary file 1 — Additional file 1: Figure S1. The expression of RND1 in normal tissues and different tumors. (A) The relative mRNA level of RND1 in normal human tissues. The expression profiles were downloaded from the HPA database. (B) The specificity of RND1 antibody was verified by western blot analysis. The anti-RND1 antibody did not show the non-specific reactions with other RND members, such as RND2 and RND3. (C) The expression of RND1 in four human GBM cell lines (U87, A172, U118 and U251) was analyzed by western blot assays. RND1 was relatively highly expressed in U87 and A172. (D) The expression of RND1 in different tumors according to TCGA database. [file 13578_2022_791_MOESM1_ESM.tif]

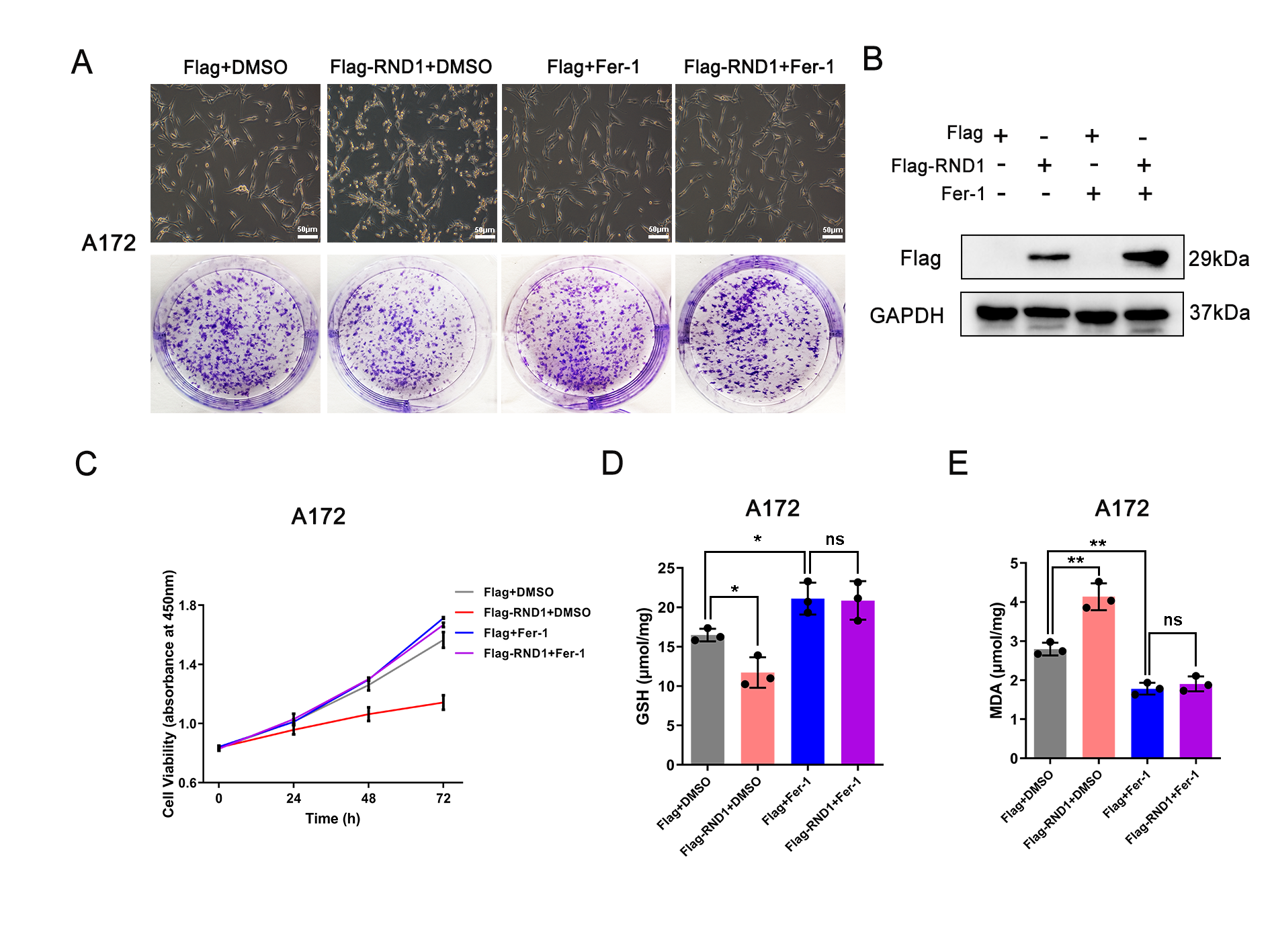

Supplement: Supplementary file 2 — Additional file 2: Figure S2. RND1 overexpression induced the ferroptosis in A172. (A) RND1 suppressed the growth of A172 and reversed by Fer-1 according to the inverted microscope observation and colony formation assay. (B) A172 cells were transfected with Flag or Flag-RND1 plasmids and treated with Fer-1 (10 μM). (C) Cell viability assay showed that the duration of Fer-1 (10 µM) significantly inhibited the effect of RND1 overexpression in A172. (D-E) The glutathione assay and peroxidation assay revealed a regulation of RND1 on the GSH and MDA level of A172 cells, which was reversed by Fer-1 (10 µM, 48 h). *, P < 0.05; **, P < 0.01; ***, P < 0.001; ns, no significance. [file 13578_2022_791_MOESM2_ESM.tif]

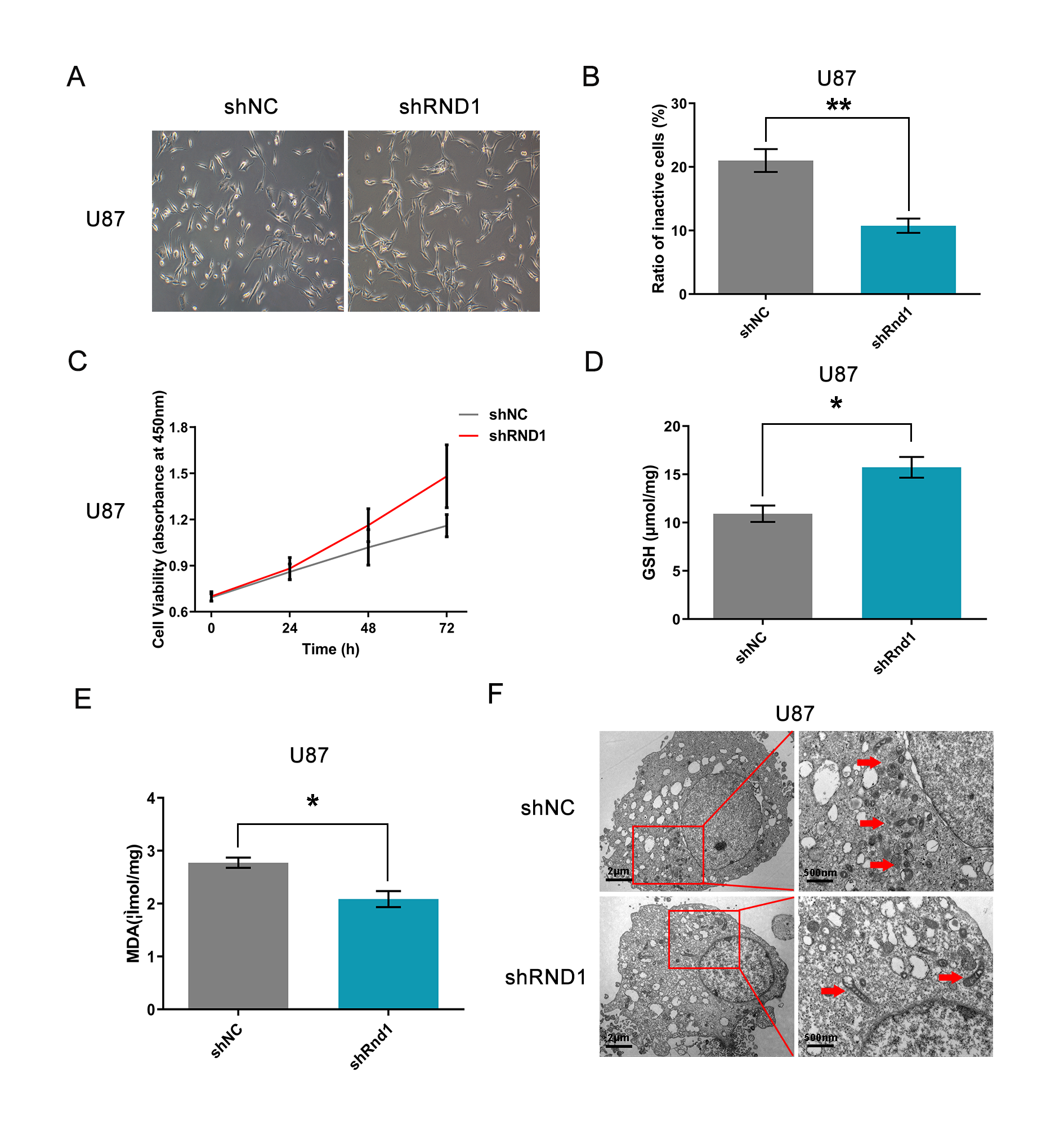

Supplement: Supplementary file 3 — Additional file 3: Figure S3. RND1 knockdown promoted cell growth and inhibited ferroptosis in U87. (A-B) Effects of RND1 knockdown on U87 cell growth according to the inverted microscope observation. The inactive cells were quantified. (C) The cell viability assay showed that RND1 knockdown promoted cell growth. (D-E) The glutathione assay and peroxidation assay revealed a regulation of RND1 on GSH and MDA level of U87 cells. (F) Transfected U87 cells were prepared for transmission electron microscopy observation. *, P < 0.05; **, P < 0.01. [file 13578_2022_791_MOESM3_ESM.tif]

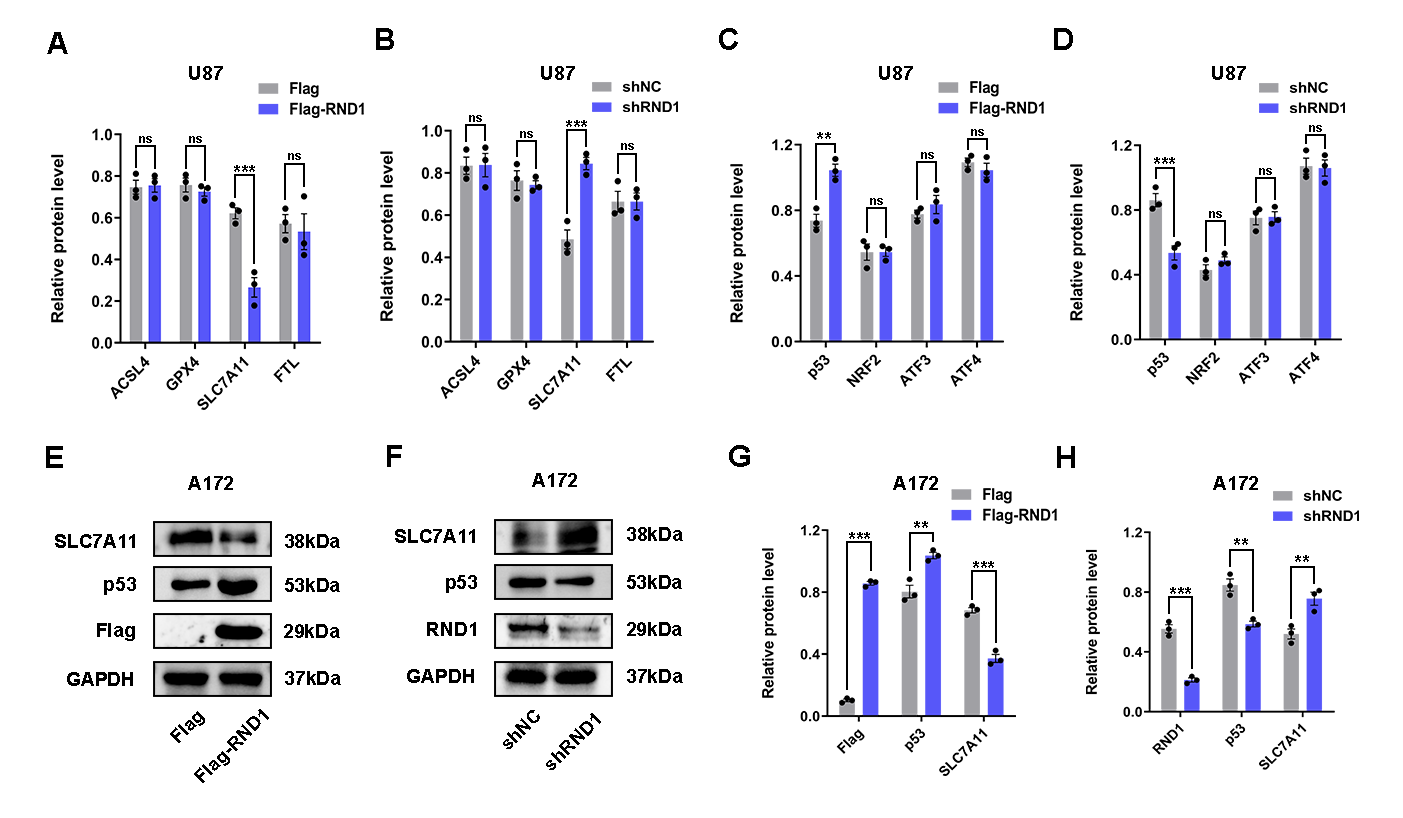

Supplement: Supplementary file 4 — Additional file 4: Figure S4. RND1 downregulated SLC7A11 and upregulated p53 in vitro. (A) The protein levels of SLC7A11, GPX4, ACSL4 and FTL were analyzed when RND1 was overexpressed in U87. (B) The protein levels of SLC7A11, GPX4, ACSL4 and FTL were analyzed when RND1 was knocked down in U87. (C) The protein levels of p53, NRF2, ATF3 and ATF4 were analyzed when RND1 was overexpressed in U87. (D) The protein levels of p53, NRF2, ATF3 and ATF4 were analyzed when RND1 was knocked down in U87. (E–F) According to western blot assays, RND1 downregulated SLC7A11 and upregulated p53 in A172. (G-H) The protein levels of SLC7A11 and p53 were analyzed when RND1 was overexpressed or knocked down in A172. [file 13578_2022_791_MOESM4_ESM.tif]

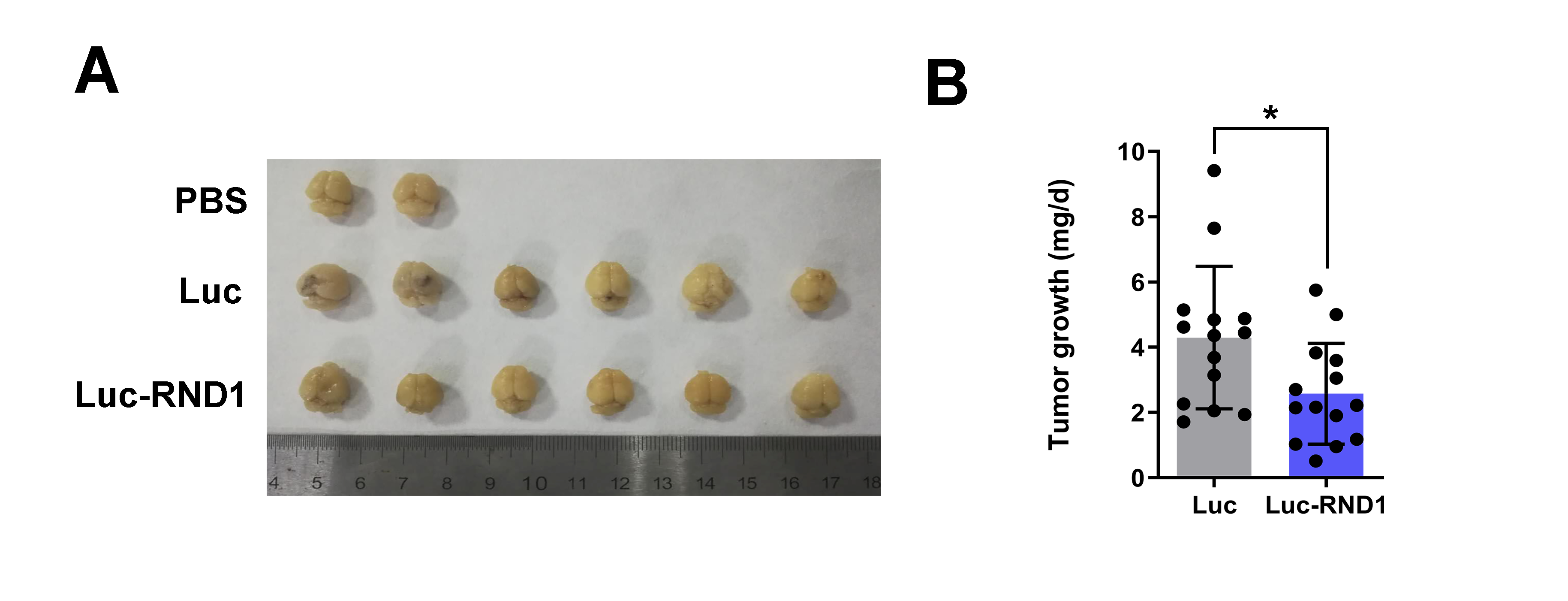

Supplement: Supplementary file 5 — Additional file 5: Figure S5. RND1 suppressed the growth of GBM in vivo. (A) Representative images of mouse brains in intracranial xenograft models. (B) The growth of tumor in xenograft models was analyzed as tumor weight /survival. [file 13578_2022_791_MOESM5_ESM.tif]

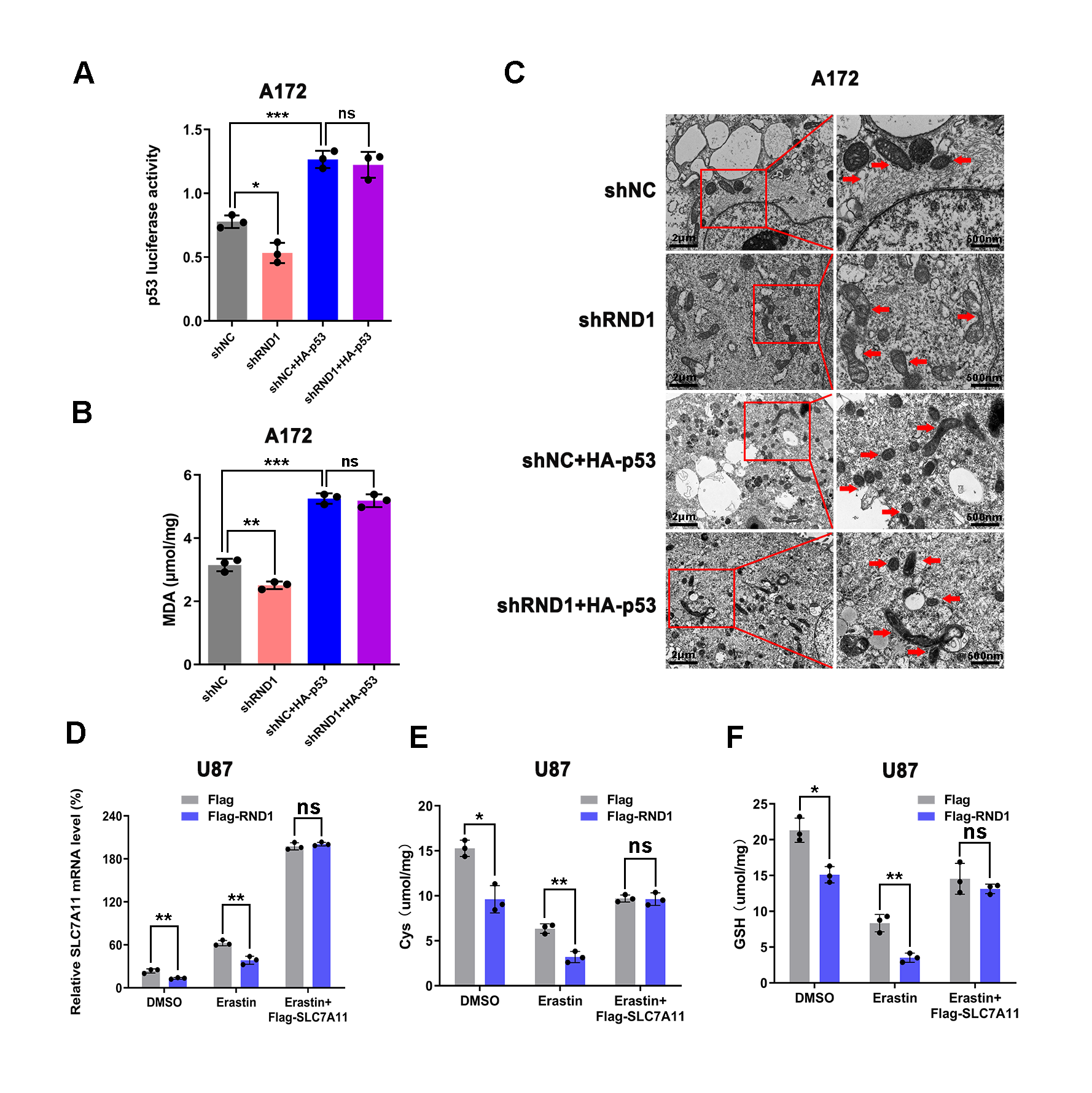

Supplement: Supplementary file 6 — Additional file 6: Figure S6. p53 acted a key role in RND1-induced ferroptosis in GBM. (A) Knockdown of RND1 significantly decreased the p53-driven luciferase activity in A172, which was rescued by HA-p53. The luciferase activity was calculated as Firefly luciferase/Renilla luciferase. (B) Knockdown of RND1 significantly inhibited the MDA level in A172, which was reversed by HA-p53. (C) Trough the observation of transmission electron microscopy, knocked down of RND1 suppressed the ferroptosis of A172, which was reversed by HA-p53. (D) RT-PCR showed that SLC7A11 was upregulated with Erastin stimulation and downregulated in the RND1 overexpressing cells. (E–F) Cys and GSH assays showed function of SLC7A11 in the regulation of ferroptosis by RND1 upon Erastin stimulation. *, P < 0.05; **, P < 0.01; ***, P < 0.001; ns, no significance. [file 13578_2022_791_MOESM6_ESM.tif]
